# Selective patient and public involvement: The promise and perils of pharmaceutical intervention for autism

**DOI:** 10.1111/hex.12637

**Published:** 2017-10-31

**Authors:** Ginny Russell, Sandy Starr, Chris Elphick, Raffaele Rodogno, Ilina Singh

**Affiliations:** ^1^ Medical School/College of Social Science and International Studies University of Exeter Exeter UK; ^2^ Progress Educational Trust London UK; ^3^ Department of Philosophy and History of Ideas Aarhus University Aarhus Denmark; ^4^ Department of Psychiatry and Oxford Wellcome Centre for Ethics and Humanities University of Oxford Oxford UK

**Keywords:** autism, biomedicine, patient and public involvement, PPI

## Abstract

**Background:**

Guidelines suggest the patient community should be consulted from the outset when designing and implementing basic biomedical research, but such patient communities may include conflicting views. We examined how engagement occurred in one such instance.

**Objective:**

Our objective was to scrutinize patient and public involvement (PPI) by a pan‐European biomedical consortium working to develop drugs to treat autism. We aimed to use this as an example to illustrate how PPI has been utilized in biomedical research.

**Setting, participants and analysis:**

Two public events, one in the UK and one in Denmark were conducted as part of the consortium's on‐going PPI activities in 2014 and 2015. Sixty‐six individuals submitted written comments on the consortium's research after these events. The textual data produced were analysed using a thematic approach. Approximately 71% of respondents reported themselves to be adults on the autism spectrum or parents of children with autism.

**Results:**

The themes identified illustrated major differences between some community concerns and the biomedical research agenda. While treating autism per se. was seen as problematic by some, treating specific co‐occurring problems was seen as helpful in some circumstances. The biomedical consortium selected PPI with a limited user viewpoint at its outset and more widely once basic research was on‐going.

**Discussion:**

This case illustrates what we term “selective PPI” where only a sympathetic and/or limited patient viewpoint is included. Findings highlight the perils of using selective PPI to legitimise scientific endeavours, and the possibilities for constructive dialogue.

## 1 INTRODUCTION

While there is much research on engagement with patient communities in health services and clinical research, less attention has been paid to the role of patients in shaping biomedical research agendas.^1^ Patient and carer engagements are recognized as an important component of drug development and implementation. Patient and public involvement (PPI) aims to improve the quality and responsiveness of biomedical research to public expectations and needs, and to influence clinical guidelines.^2^ PPI is now a central feature of biomedical research applications and is required by funding programmes.^3^ The rise of PPI as a central tenet of biomedical research has been supported by social science research pointing to the value of lay‐expertise and local knowledge situated in context and experience.^4^, ^5^ Patients should be involved in biomedical research development for ethical, political, and pragmatic reasons: In the first case, it is the moral right of patients to influence discussions that will affect their lives; in the second, the accountability of public money is at stake; and in the third, lay‐knowledge can improve translation of interventions to user communities more effectively.^6^ PPI is encouraged at every stage, from generation of research questions through to dissemination, so that basic biomedical research can translate into what is useful to, accessible to, and welcomed by patient communities.

Despite this policy directive to address patient concerns from the outset,^7^ the evidence that publics and patients are involved in biomedical research is mixed.^8^ Researchers themselves sometimes view PPI as something they are required to do in order to fulfil funding guideline requirements: PPI is seen as a “tickbox” exercise for some in biomedical industry and academia.^9^ One qualitative study found patients’ perspectives were excluded during meetings with experts, even when inclusion strategies were put in place.^10^ Here, we examine an instance of PPI in biomedical research (centred on the development of pharmacological treatments for autism) to illustrate how PPI has been utilized.

Autism is a good example of a health condition that has inspired a huge amount of community, political and social activism over many decades.^11^ Many in the autism community feel they are not listened to by the research community, even when their views are solicited.^12^ Within the context of autism activism, the subject of a “cure” for autism has received a good deal of attention and divided community opinion. Two key activist viewpoints regarding a cure for autism have been described in polarized terms as: (i) a pro‐cure stance (mainly represented by biomedical researchers and some charities); and (ii) an anticure stance (mainly represented by adults with autism and some parents).^13^

Many autistic adults promote efforts to provide accommodations to remove specific obstacles for individuals with autism, but decry efforts to develop “cures.” These members of the autism community oppose elimination of autistic traits or symptoms.^14^ Their groups often deploy ideas of the disability rights movement especially the emerging neurodiversity movement, which sees autism and other neurodevelopmental disorders as a natural part of human variation, rather than a disorder. The movement is informed by the social model of disability that has traditionally separated personal impairment from disabling aspects of society.^15^ Here, disability is regarded as an emergent characteristic of individual differences encountering inaccessible or discriminatory social environments.^16^ Thus, the onus falls on society to change; that is, to embrace and accept autistic traits and behaviours, and act to accommodate them, rather than target individuals with autism for “cure.” In a thoughtful analysis of online discussions, Brownlow has described how individuals with autism are constructed by medicine as needing to change (via intervention and treatment) in order to accommodate the non‐autistic world.^17^

The subject of autism treatments has received less discussion than the more provocative and dichotomous subject of autism cure. Numerous groups around the world are conducting research in the biological substrates of autism, in a stated effort to develop more effective treatments.^18^ Clinicians and biomedical researchers anticipate that basic biomedical research encompassing genetics and neuroscience may eventually lead to new and more effective drug treatments for autism.^19^ The goal of autism treatment, according to medical professionals, is to improve communication, social, adaptive, and behavioural skills which constitute the core symptoms of autism.^20^

Autism treatment development is backed by relatives’ collectives and charities, including *Autism Speaks*, who argue that, “medicines for treating the three core symptoms of autism – communication difficulties, social challenges and repetitive behavior – have long represented a huge area of unmet need”.^21^ Charities in the USA are the leading private funder of biomedical research in autism treatments; the charity *Cure Autism Now* provided more than $39 million for research grants before merging with *Autism Speaks*. Within the protreatment community, parents of younger and severely affected children speak passionately of the importance of efforts to develop biomedical treatment for what they see as a highly impairing and distressing condition.^22^ There is currently insufficient evidence to evaluate the benefits or adverse effects for pharmaceutical treatments for autism,^23^ and pharmacological interventions are not currently recommended by national or international guidelines.^24^

As is evident from this overview, the autism community should not be considered as one homogenous voice, just as the public cannot be considered to share the same views. Within the autism community there are multiple “publics”^25^: small groups of people who follow a particular issue in detail. They are well informed about the issues and have very strong opinions on them. Given the necessity of PPI in basic biomedical research, it is important to establish what is and what is not acceptable to the various factions in the autism community regarding drug development, particularly given that at time of writing there were over 40 on‐going clinical intervention trials registered with the US National Institute of Health testing drugs to treat autism (Clinical trials.gov). As the trend to diagnose more children with autism continues, developing a pharmaceutical treatment for core autism symptoms in childhood could become an attractive target for further pharmacological industry investment. We were interested in how opinions on drug treatment varied in the autism community and whether the biomedical consortium had involved different patient and public opinion in its decision making. Our research questions were (i) what were patient and community views concerning development of pharmacological treatment for autism, and (ii) to what extent were these taken into account by the biomedical scientists in shaping their research agenda?

## 2 METHODS

### 2.1 Background

In 2012, a large pan‐European consortium, known as EU‐AIMS, was funded to contribute understanding of underpinning biological mechanisms of autism and to leverage this knowledge to develop effective pharmacological and other interventions for autism. PPI was woven into consortium activities from the start, with a parent‐run charity that is a known supporter of treatment of autism (*Autism Speaks*) as a co‐applicant on the grant. The consortium has an independent ethics advisory board (EAB), of which most of the authors are members.

In 2014 and 2015, the EAB convened two public events, one in London in the UK and one in Aarhus in Denmark. Both events were advertised widely via Universities and EAB networks, and via networks in the autism community.

During these two events, a promotional video was used as a prompt to solicit comments about the consortium project agenda. The video is freely available online, accessible at the bottom of the front page of the EU‐AIMS website, branded “EU‐AIMS project—What is EU‐AIMS doing for autism research?”.^26^ A transcript of this video is available in the supporting material (Video S1). The video consists of interviews with prominent scientists working on individual biomedical studies that make up the consortium. These are upstream research projects focussed on discovering biological predispositions to autism and turning this knowledge into treatments. Primary areas of investigation include genetic markers of autism, mouse models, and brain‐based biomarkers. The video describes the ultimate aim of the project as being the effective harnessing of these approaches towards the production of pharmacological treatments for autism. Our aim was to assess diversity of community views to illustrate how PPI was used in the biomedical context. We analysed these data and report the extent to which community feedback was taken into account by the consortium.

### 2.2 Data collection

The video was used as a prompt to solicit comments (our data) about the research agenda. Data were collected in 2 waves. Wave 1 was after the public events in 2015, EU‐AIMS where a call for open comments on the video was made at the PPI events themselves. Wave 1 data were collected through emailing everyone who had left contact details at or before the events, inviting them to submit open comments about the video prompt. This included an autism charity that attended, and three autism groups. Wave 2 data were collected after a request from the consortium was made to diversify the sample (after comments that the sample was biased towards cognitively able self‐advocates). As qualitative data, the responses represent a snapshot of opinion of a mobilized group from within the autism community, and we do not claim that this group is representative of the autism community as a whole. Nevertheless, a second wave of data collection took place in 2016, via a free‐response online survey. This was intended to diversify the initial sample, albeit within the constraints of a qualitative survey. The free‐response survey was linked from the EU‐AIMS cohort Facebook page, and linked to the video prompt which was hosted by the EU‐AIMS website. The online survey is available as supporting material. Seven additional responses were obtained at wave 2.

These activities generated a data set consisting of approximately forty pages of written comments on the video prompt, from 66 individual respondents. We asked respondents to report their relationship to autism and classified them as either having a diagnosis themselves, being a family member of a person with autism, having a professional interest or without any relationship. As most respondents who declared this information had autism themselves, were directly related to a person with autism, or worked with children or adults with autism, we refer to the sample as “the autism community.”

### 2.3 Ethics

Ethical approval for this study was given by the University of Exeter Ethics Committee at the school of social sciences: approval reference: 201516‐063. Contributors who submitted comments were provided with an information sheet about the current study detailing its rationale, and how their contributions would be reported. All respondents quoted and analysed here gave their explicit consent for their contributions to be included after reading this information.

### 2.4 Analysis

Data were analysed using a thematic approach to identify the significance of patterns and their broader meaning. The method is described in detail elsewhere.^27^, ^28^ First, two researchers familiarized themselves with the data by reading and rereading the initial data set and noting early ideas. Second, preliminary codes were generated from cataloguing use of certain words and descriptive phrases in the context of the intended meaning across the entire data set. The original words used in the data were used to define the meaning of each code. Thus, the coding phase retained the original meaning as much as possible. Codes were cross‐checked between two researchers, and a coding frame was agreed. The complete data set was then coded using NVivo software.

Codes were collated into potential themes, gathering all data under each code relevant to each putative theme. Themes were reviewed to check they made sense in relation to the coded extracts and with the entire dataset. Counter examples to the overall narrative were coded and included. Finally, appropriate quotes were selected to reflect the overall interpretation in the analysis.

## 3 RESULTS

The participants’ relationship to autism was declared in the bulk of cases (n = 41). Of these, around half n = 20 (49%) were adults with autism, 9 (22%) were family members of people with autism (almost all were parents), and 8 (19%) were involved with autism in a professional capacity, for example teachers, clinicians, researchers and nurses. Most respondents were critical, probably because people who were motivated to respond did so to question the status quo on the development of drugs for autism. The two major themes identified are outlined below.

### 3.1 Theme 1: autism is not a suitable target for drug treatment

The promotional video described autism as a “disease,” which provoked a strong negative reaction from many respondents. Many were uneasy with autism being described as having a biological cause, pointing out there were diverse developmental pathways to autistic phenotypes, including “the attitudes of other humans.” Respondents thought that a disease label for autism was wrong:

It (the video) made autism sound entirely negative, like a disease. Autistic adult A

What on earth is Professor X doing calling autism a ‘disease’? It's not a disease. Family member A

Analysis from the USA shows the majority of funding for autism research is currently for basic biomedical research.^29^ But autism, it was argued, was not suitable for pharmacological treatment in the same way as a short‐term disease such as pneumonia, as “curing” was equated with changing somebody's identity.

Autism is such an inseparable part of our identity, and eliminating it (if that is even possible) would mean eliminating the individual. Autistic adult C

The heterogeneity of the condition and its nature as a spectrum “encompassing both the severely disabled and remarkably gifted” (in some cases a person may be both) led participants to question whether one treatment for all individuals was desirable or possible.

To what extent is it possible to talk in global terms about treating autism, given the wide heterogeneity of the condition and given the existence of disparate comorbidities? Parent A

Overall, many participants were sceptical about drug development, questioning whether and in what ways treating autism with pills would enable children and adults to flourish.

Are some scientists trying to “make” certain types of human beings by eliminating parts they don't like? Who will decide on the “treatment” if/when it is put in place; will it be doctors, parents/carers or autistic people themselves?

By “eliminating” autism or autistic traits we are losing a lot of important and useful features that these traits contain. Autistic adult D

Participants clearly adopted narratives of the neurodiversity movement that are directly related to social models of disability, but viewpoints were often nuanced with recognition of both strengths and challenges. It was acknowledged that people with autism might need help and support, but many also recognized advantages of autism that were in danger of being lost should a drug treatment be prescribed. Given these nuances, there was a strong resistance to the idea of “blanket approach” drug treatment, particularly from autistic individuals themselves.

I think the use of drugs to eliminate autism would be a terrible idea, as it would eliminate large parts of the person's character and if done on a large scale, would make the world a poorer and less diverse place. Autistic adult E

A mother wrote about her son who had severe autism at age three, but celebrated his later achievements (as an adult). She noted how appreciating his adult accomplishments might not have been possible had drugs been prescribed when he was small, because treatment might have dampened some of his autistic traits:

If his doctor had said at the time that a drug was available to make him more sociable and make him like other children, and if his doctor had recommended we try this drug, I would probably have done so. In hindsight, I am enormously glad that such a drug was not available. Parent D

The point was that autistic traits could not be treated in isolation. If challenging aspects of autism were treated, this could result in the loss of valuable aspects too, throwing the baby out with the bathwater, so to speak.

In contrast to these views, some parents of severely affected children were in agreement with the message that autism can be considered akin to a disease that is harmful. A number of parents expressed forceful support for the drug development programme. These parents thought that, if there were drugs available, they would be used in combination with existing behavioural therapies and that this would improve their children's lives and provide needed support. One parent described his hopes for treating his son who was diagnosed with infantile autism:

If my son could take a pill for his autism and thus get rid of all the problems he contends with and is going to fight with, I would almost force‐feed him with such a pill! I cannot understand the desire to ‘protect’ a handicap for handicap's sake! Parent B

These voices were reminiscent of those of parents of younger and severely affected children elsewhere who have spoken fervently about the significant need for research to develop biomedical treatments for what is, for them, an extremely damaging and demanding disorder.^22^ Most comments that were supportive of drug development came from parents of young and severely affected children suggesting this group are more likely to be end users of a pharmacological treatment.

### 3.2 Theme 2: circumstances when drug treatment is acceptable

Circumstances under which drugs would be acceptable and/or welcomed were described by some adults who were themselves on the autism spectrum. These involved short‐term gains, and control by the person taking the drug, and did not involve “autism” as a generic target.

Agency was an important subtheme of drug treatment acceptability. Many participants stated it would be acceptable to treat if the individuals wanted it, and the person him/herself was able to control how, when and why the dose was administered. If the individual was coerced or did not wish to be treated, it was unacceptable to administer a drug. Simply put, “you should not treat someone who does not want to be treated.” Control and agency, it was noted, are particularly difficult to achieve in the case of individuals with an intellectual disability or in those who are minors, and this led to heightened concerns about enforced treatment in these cases. Some respondents asked the question: “who decides”? in the event of an anti‐autism drug coming to market. Participants were worried about who would determine when treatment should be administered, to whom treatment should be administered, as well as when it would be deemed a success.

How do researchers decide when a child should be given medication and if it will be in the child's best interests? Parent C

Respondents went beyond critique of biomedical research priorities to question whether the products of research would translate into ethical clinical and societal practices. The use of psychotropics as a convenient form of social control, to suppress difficult or unwanted behaviours, was a concern. A Swedish study found autistic adults’ own agency was needed to render intervention meaningful to them.^30^ Other participants pointed out that drugs might be used as a matter of convenience, due to a combination of time pressures on clinicians and parents. One mother illustrated how she felt the mix of medical advice and time‐pressure had led to a prescription culture.

When my son was a child and adolescent, a lot of his behaviours were attributed to his autism, when ..he was in excruciating pain from several debilitating medical conditions. ….The point is, I am very worried that if there were blanket drug treatments for autism, he would have been put on these, which would have been a completely inappropriate red herring… If blanket drugs become available, I imagine clinicians ‐ hard‐pressed, busy, overworked etc ‐ will automatically reach for the prescription pad, rather than exploring more fully what the underlying issues are. Parent D

Two participants expressed concerns over the involvement of pharmaceutical companies, suggesting this could lead to publication bias towards positive findings, or become a market‐driven process where the ultimate aim was to sell as much of the pharmacological product as possible.

By contrast, some autistic adults saw a drug as a potentially useful tool to be used as required to manage stressful situations, or a way to deal with co‐occurring problems such as anxiety, epilepsy, or other forms of distress. One adult with autism gave six specific examples of when and how drugs might be helpful, but only in the case where the person could decide when and how much of the drug was taken. This is one of her suggestions:

A way of “knocking one's system into gear” when immobilised – something like a piece of nicotine gum or a cup of strong coffee or some dextrasol, but specific enough to take on autistic stuckness. This would need to be very easy to access at all times. Autistic Adult E

Finally, there was a call for greater involvement of the autism community in future biomedical research. The charity *Autism Speaks* also came in for criticism, with autism community members questioning its “alarmist rhetoric,” and the lack of representation of people with autism among employees and on the Board of Trustees. Overall it was clear there was more work to be done by the research community in terms of community engagement: “There needs to be greater involvement of the autism community in targeting resources to the critical needs of people with autism and their families, and in translating research into practice,” as one parent put it.

To summarize, respondents had a diverse range of opinions, ranging from those who raised concerns about biomedical research effort to drive the development of drugs, and its fundamental assumption of the need for treatment and how this positioned autistic individuals, whilst others wrote about the circumstances where drugs would be acceptable and/or welcome for them, through to a small group of enthusiastic advocates of the biomedical research. Respondents also wrote about concerns over the use of drugs in practice, and the importance of including a range of patient views.

A summarized list of the respondents’ comments was submitted to the consortium in 2015, and presented at a consortium meeting in 2016. The consortium decided to publish written replies to this material on its website. At the time of writing (summer 2017), publication of these replies drafted by members of the consortium scientific community had stalled, however the EAB had filmed a series of interviews with members of the consortium addressing community views, which the scientists addressed seriously and in good faith. The consortium agreed to feature the film on their website going forwards.

## 4 DISCUSSION

The findings show the autism community is an example of a patient community which holds diverse opinions about the promises and perils of biomedical research. Biomedical researchers initially selected community voices that were known to support their agenda (to develop drugs to treat autism) when developing their grant, through a partner Autism charity which itself funds drug development. At that time, the consortium did not engage with those members of the community that did not share these views. Our findings led us to define and illustrate the concept of *Selective PPI*, where only a sympathetic and/or limited patient viewpoint is included. The analysis demonstrates the need for major biomedical research projects to consider whether their PPI strategy might be selectively co‐opting community voices that uncritically support a biomedical agenda. Below, we consider the mismatches that acted as barriers to meaningful patient and public involvement in this case.

### 4.1 Barriers to participation: mismatches

In their 1998 review of the PPI literature in health research, Grant‐Pearce, Miles & Hills define “mismatches” as differences or disagreements in perception, opinion, view or practical decision concerning the problem or needs to be addressed by research. They noted that these mismatches were under‐recorded, and that very few studies in their review were able to draw direct comparisons between priorities of patient groups and professionals.

One mismatch exposed in our study was due to different ways “autism” was constructed by different actors, in line with their own agendas. In particular, there was tension between a “medical” vs a “social” model of autism. These models represent polarized perspectives on how autism is experienced and understood across different cultural contexts.^31^ Millions of dollars in funding and investment have been based on the biomedical narrative of autism.^29^ Social scientists have noted interventions unintentionally but intrinsically cast target behaviours as undesirable^32^, ^33^ (a point applicable to all forms of childhood behaviour that are deemed treatable, for example, inattention and hyperactivity in ADHD^34^). The video prompt utilized a medical model to cast autism as a disease; that is, as a discrete and undesired entity separate from the individual. Pharmacological intervention promised to decrease the suffering caused by disease. However, many respondents in our study, particularly adults with autism, viewed autism as an identity. These respondents questioned the implicit paternalism in autism intervention, arguing that intervention should not be assumed to be necessary. Thus, the video unwittingly created a barrier between the research consortium and the autism community by assuming that intervention was required for people with autism; by objectifying autism as an entity apart from the person; and by commodifying the contributions of autism research participants in an (identity‐destroying) pharmacological product, all recognized as barriers to participation in autism research.^35^

A second mismatch was one of timing. As a collaboration between the Innovative Medicines initiative (IMI), the European Commission (EC) and the consortium institutions, EU‐AIMS had an obligation to include PPI in the research project. PPI guidelines^36^ stress that researchers should involve users from the outset of the research. As noted above, the charity *Autism Speaks* was included as a formal collaborator on the project; it has helped to identify research priorities, and it had membership on the consortium advisory team. However, *Autism Speaks* represents a particular view on biological treatments for autism (strongly in favour) that is not shared by others in the autism community. Where there are intracommunity patient debates, certain positions may be co‐opted to support a particular research agenda. When the consortium first presented the research project to the broader autism community, and put up the video, was about 2 years into a 5‐year research project. Thus, there was a mismatch in the timing of the start of the research project and the timing of the broader autism community's invited engagement. By the time of our community survey, the biomedical consortium was already committed to pre‐specified work to target behaviours listed as core autism symptoms by agreement with funders. Thus, the later PPI was rendered “toothless” in that the biomedical research agenda was already set. The biomedical scientists’ responses were promised, but this process was stalled several times: at this point community concerns appeared to be seen as obstacles to be navigated, rather than utilizing them to set a research agenda. Addressing community antipathy may require careful coproduction from the outset.^37^, ^38^

We therefore argue that the involvement of the charity *Autism Speaks* as the sole representative of the autism community in the research consortium is a case of “selective PPI,” in which PPI guidelines were followed by the biomedical consortium to the letter,^36^ but in a way that involved only those community voices that supported the consortium's research agenda. While the involvement of *Autism Speaks* gave the research endeavour PPI direction and legitimacy at the point of submission for funding, it also ensured that, once the more critical voices of the broader autism community were engaged, their views would not alter the research agenda.

It is worth considering what “inclusive PPI” would have meant for the consortium research, and who is responsible for ensuring that inclusive PPI is undertaken prior to submission for funding. While the project protocol was still under construction. Inclusive PPI would almost certainly have prolonged the development of a highly complex, multinational research project. In large research consortia, pre‐submission negotiations among researchers and with funders and industry require upfront investment of time and resources. The infrastructure of biomedical funding does not require a range of patient views, just patients’ views. It is not clear who should take responsibility for ensuring an inclusive PPI process at this point in the research cycle; however, we do not believe that the responsibility rests with the biomedical researchers alone. PPI is a higher‐level mandate sent down from funders and policymakers, but it not part of the core business of most researchers. Moreover, the increasing emphasis on industry‐academic partnerships in research pulls against inclusive PPI. The expectation of a viable commercial product to justify investment means that, in some cases, arguments against research that would produce such a product will be marginalized. Of course, this does not mean that such arguments should not be heard in the first place, nor does it mean that such arguments should play no role in shaping the research protocol.

Selective PPI is enabled because, despite the rhetorical emphasis currently placed on PPI in research, there is no identified role in a biomedical project team in which responsibility for fair, just and inclusive PPI from the start of the research cycle resides. PPI plans are also not routinely reviewed by an independent PPI expert following submission of the grant. The responsibilities—for insisting on a PPI role from the outset of project development and an independent expert review of the PPI plan—rest with funders. As research funding procedures are dominated by academics, and funding is based on scientific criteria, with biomedical research plans specified at the outset, is it surprising that many biomedical scientists currently see PPI as a tick box exercise? Arguably too, if inclusive PPI is a higher‐level mandate, there should be a great deal more useful and structured training for researchers, training that includes analysis of mismatches and conflicts such as the ones identified here.

## 5 CONCLUSION

The findings stress the importance of involving a range of patient views in research from the outset: from the generation of an idea through to research design and use of any outputs in the clinic and community. This is seen as a moral obligation to ensure that biomedical research addresses the needs of the user and carer communities, as research translates from bench to bedside.^39^ Thus users or patients are accorded “privileged knowledge”—a person carries more epistemic authority because they are from a certain group. Our case study raises the question of how PPI should take into account contrasting positions from within this patient community. It highlights what others have noted: that current strategies for patient participation in biomedical research decision making are not effective.^1^ It also highlights the practical moral perils of using what we call “selective PPI” to legitimise scientific endeavours; and the enormous challenges of properly taking the views of a heterogeneous community into account within an international research portfolio that involves multiple funders, institutions and organizations.

## CONFLICT OF INTEREST

None declared.
