# Supplementary material for: Selective patient and public involvement: The promise and perils of pharmaceutical intervention for autism
Source: Health Expect. 2017 Oct 31;21(2):466–73. doi: 10.1111/hex.12637 (PMC5867326; doi:10.1111/hex.12637)
Supplement: Supplementary file 1 [file HEX-21-466-s001.docx]

**Video prompt transcript**

**What is EU-AIMS doing for Autism?**

https://www.youtube.com/watch?v=xXuxi7dfVn0

(.) – Pause

(…) – long pause

**Interviewee 1**: The EU-AIMS was born out of the idea that we want to develop new drugs for

autism disorders (.) um soon enough we realized that we could not do this alone (.) we needed a host of key stakeholders these include pharma people it includes academic people

and indeed includes *(EMA? Muffled)* people (.) after that we were realizing it can only be done through an enormous project.

**Interviewee 2:** the reason that academics became involved in this was because autism is such a huge problem for Europe (.) about 1 in100 people are affected by autism and there are no effective treatments at the moment for the core symptoms of autism (.) therefore we needed to identify new treatment targets and work together with our colleagues in industry to make that happen.

**Interviewee 3:** well so the stem cell part of EU-aims is built on a Piece of technology that's pure magic (.) so what we’re able to do now is to pluck just single hair from an autistic patient or a neurotypical patient (.) and from the root of that hair we can grow a population of cells and then using a piece of molecular magic we can transform those ordinary hair keratin sites into true stem cells (.) and these stem cells we can then use to differentiate into any cell type we like (.) any cell type in the body (.) including of course brain cells and that allows us then to do a really exciting experiment because what we can do is we can grow those cells develop those brain cells in a culture dish (.) and we can compare how the cells from an autistic individual grow compared to those from a neurotypical individual (.) and that way we hope to be able to get an insight into what causes the disease and what brings it about.

**Interviewee 4:** so one of the key challenges for the EU-AIMS' project is to develop and identify the best animal models of autism and this is to help both with basic discovery and and drug discovery and drug development efforts (.) so two of the things that we focus on are to answer the question of what is the best animal model of autism (.) one of these is face validity (.) meaning that we want to recapitulate some of the behavioural symptoms that we see in humans in in rodents mice and rats and fortunately mice and rats are social creatures they exhibit things like repetitive behaviours they can have better or worse memory in many of the symptom domains that we find in autism (.) the second thing is that we have very unique methodologies and techniques to to mutate some of the genes that have been shown to be contributing are causal in autism spectrum disorder so we can make very fine mutations in key genes and this helps to establish the construct validity for those models.

[3.00]

**Interviewee 2:** the next thing that we want to do is to translate all that amazing work from stem cells and rodent models into people because that's who we're trying to help (.) the other thing that we need to do is to train the next generation of research leaders so what we have is some just fantastic PhD students who are leading some of the experiments that we’re carrying out and one of those is Laura and she’ll be speaking about what she's been doing

**Interviewee 6 :** I have the really exciting job of translating the work from my colleagues in the lab into the human studies in the clinic (.) this is really exciting at the moment because not only have we been able to replicate the studies in the lab in humans but also we’re able to use this information to make real headway into the development of new drugs for autism (.) as a student as part of the EU aims we have access to both training in clinical and basic science from world leaders and this is really important and valuable to career progression

[4.00]

**Interviewee 1:** We are also conducting a set of extraordinary and unprecedented clinical studies in EU-AIMS one of these is an inference that there is a high familial rates for autism because they have older brother or sibling with autism (.) in the siblings we assess brain function cognition we assess behavioural qualities and so we are able to identify biomarkers and mechanisms that may proceed the onset of clinical symptoms

**Interviewee 8 :** we’re also running studies with patients with monogenic forms of autism (.) so we estimate currently that about 10 in 10 or 20 percent of patients with autism the condition is caused by a single gene (.) now this is potentially very important for two reasons firstly it offers us a new window of opportunity to identify treatments not only based on a person’s symptoms but based on what actually causes the conditions particular symptoms

[5:10]

**Interviewee 9:** so one other thing we're doing put as part of the EU-AIMS is we're trying to develop uh very large clinical networks at the moment we have over 80 sites from over 35 countries who um are jointly collaborating with us on trying to bring together some of the clinical datasets that that exist in Europe and people sharing data with us and we hope to find out new things from having very large data sets that are pooled together from all the clinical sites that are working on those studies (.) Sandy we wanted to ask you about your views from an ethics perspective around the work that's going on within the EU-aims consortium

**Interviewee 10:** well I think all of the research that's taking place under the auspices of EU-AIMS is based on two fundamental assumptions that not everyone will agree with the first is the assumption that autism is biologically explicable and the second is the assumption that autism is amenable to treatment medical or otherwise (.) it's very important not to take for granted that people will agree with these assumptions and to keep making the arguments a new for why these assumptions might be valid it's also very important for EU-AIMS to talk to and keep talking to people with autism their families their carers and their supporters so that at the very least they can understand what research is taking place and why and researchers can understand how autism is viewed and what the ethical ramifications of their research might be

**Interviewee 1:** now three years in the project it's great to see how all these different stakeholders work together towards a new goal and this goal is to obviously to develop a drug the drug discovery will take place in the company's there will be a time before it’s on the market but for sure EU-AIMS has made great contribution to the success

**Interviewee 2:** in only the last three years we've made tremendous progress my colleagues doing basic science have discovered new molecular mechanisms that might explain a proportion of autism and they've shown that those deficits can be reversed we’ve also shown within human studies that we can change brain function what we need to do is to take that into the clinic and show that those new approaches also change behaviours for the better and those approaches are acceptable to people with autism

**On-line free text survey text.**

**Page 1**

Our aim is to describe the responses of the autism community to developing pharmacological treatments for autism. Your responses will be confidential and anonymous. There are four questions. These are free text boxes so you can say as much as you like.

Here is how we will use the response you give- just to keep you informed. Please consent to participate by typing your full name on the sheet provided (link to information and consent sheet).

**Page. 2** Developing drugs to treat autism- video link

Please watch the short video on the work of ‘EU-AIMS’ here- (link).

1) What issues did the video raise for you? Reminder: The link to the video is here- (it is at the bottom of their page) Please write as much or as little as you like about any issues the video raised for you.

**Page 3**. 3 About you

Preview question

2) What is your relationship to autism ?

- you have a diagnosis of an Autism Spectrum Disorder
- your child has a diagnosis
- other

a if other please specify

3) What is your gender

Male

Female

Other

**Page . 4** Finally...

4) Anything else you’d like to add?

If you are happy for our project to contact you about future research, please add your e-mail address here. We won’t use it for any other purpose.
